# An Update in Guillain-Barré Syndrome

**DOI:** 10.1155/2014/793024

**Published:** 2014-01-06

**Authors:** J. B. Winer

**Affiliations:** Queen Elizabeth Hospital, B15 2TH Birmingham, UK

## Abstract

Guillain-Barré syndrome (GBS) was first described in 1916 (Guillain G, 1916) and is approaching its 100th anniversary. Our knowledge of the syndrome has hugely expanded since that time. Once originally considered to be only demyelinating in pathology we now recognise both axonal and demyelinating subtypes. Numerous triggering or antecedent events including infections are recognised and GBS is considered an immunological response to these. GBS is now considered to be a clinical syndrome of an acute inflammatory neuropathy encompassing a number of subtypes with evidence of different immunological mechanisms. Some of these are clearly understood while others remain to be fully elucidated. Complement fixing antibodies against peripheral nerve gangliosides alone and in combination are increasingly recognised as an important mechanism of nerve damage. New antibodies against other nerve antigens such as neurofascin have been recently described. Research databases have been set up to look at factors associated with prognosis and the influence of intravenous immunoglobulin (IvIg) pharmacokinetics in therapy. Exciting new studies are in progress to examine a possible role for complement inhibition in the treatment of the syndrome.

## 1. Introduction

Our understanding of the Guillain-Barré syndrome has improved greatly over the last decade with a much clearer idea of the clinical subtypes of the syndrome and the pathogenesis of some of the rarer variants. 2016 will mark the centenary of the original description by Guillain, Barré and Strohl [1]. They described a rapidly progressive motor disorder associated with absent reflexes and a raised CSF protein in the absence of the expected cerebrospinal fluid (CSF) pleocytosis that characterised poliomyelitis. It became clear, over the ensuing years, that the syndrome varied in severity so that in its severest form it could lead to respiratory paralysis and death [2]. Acute inflammatory demyelinating polyradiculoneuropathy (AIDP) is the most frequent subtype in the Western world with a primarily demyelinating pathology and various degrees of secondary axonal damage. Acute motor axonal neuropathy (AMAN) [3] is the next most frequent and appears to be a primary axonal disorder affecting just motor nerves. Axonal variants involving both sensory and motor nerves are much rarer Acute Motor and Sensory Axonal Neuropathy (AMSAN) [3]. Miller Fisher syndrome is generally considered to be allied to GBS although it has a uniquely tight association with anti-GQ1b antibodies.

## 2. Clinical Features

GBS has an incidence of about 1/100,000 across several studies [4, 5] in a number of countries. It increases in incidence with age and there is a small predominance of males [5].

Sensory symptoms in the legs usually mark the onset of the disease followed by rapidly progressive distal weakness that soon spreads proximally. Lumbar pain is common and may represent inflammation in the nerve roots and may coincide with the breakdown in the nerve CSF barrier that allows protein to leak into the CSF. The weakness of GBS is typically “pyramidal in distribution” with ankle dorsiflexion and knee and hip flexion often severely affected and likewise the weakness in the arms is usually more severe in shoulder abduction and elbow extension. While sensory symptoms are common sensory signs are usually minor and may be limited to loss of vibration and proprioception. The significance of reduced or absent reflexes with no objective large fibre sensory loss and yet complete paralysis leads to a frequent misdiagnosis of hysteria.

Respiratory involvement may be sudden and unexpected but usually the vital capacity falls steadily and intubation and ventilation are required at level of approximately 1 litre [6]. A small number of patients develop unusual signs such as papilloedema [7] thought to be secondary to cerebral oedema and hyponatraemia [8]. Mild autonomic disturbance is seen in three quarters of patients but a few develop severe bradyarrhythmias which are recognised as a cause of infrequent death from the syndrome. Mortality in most population studies is between 5 and 10 percent [9]. The disease is monophasic with weakness reaching its most severity in 4 weeks followed by a plateau phase and then recovery. 60% of patients are able to walk unaided by 12 [10] months and the rest are left with various degrees of residual symptoms.

Three quarters of patients give a history of a preceding illness usually respiratory or gastrointestinal which may be so mild as to be completely asymptomatic. The neuropathy typically begins 7–10 days after any triggering infection. Numerous other antecedent events are described including surgery and immunisation. Most recent epidemiological surveys show the risk of immunisation triggering GBS to be very low [11]. It is estimated that the risk of contracting GBS from current influenza vaccines is significantly lower than the risk of getting GBS from influenza itself. Serological studies have shown that *Campylobacter jejuni*, Epstein Bar virus, and Cytomegalovirus are the most frequent antecedent infections. Patients sometimes continue to secrete *C. jejuni* in their stool for up to 3 months following the onset of GBS [12]. Persistent infection with CMV or EBV is very rare. A number of reports associate GBS with mycoplasma pneumonia, influenza, and varicella [13].

## 3. Pathology

Autopsy studies in GBS are rare because few patients die. Early studies reported oedema of the peripheral nerves with sparse inflammatory infiltrate [2]. Classic studies by Asbury and colleagues emphasised the importance of perivascular lymphocytes which resembled the findings in the animal model experimental allergic neuritis [14]. They postulated an immunological basis for the demyelination involving these lymphocytes and strongly influenced thinking about the cause of GBS. Electron microscopic studies of nerve biopsy have demonstrated macrophage associate demyelination. Macrophages appeared to invade the Schwann cell basement membrane and phagocytose myelin debris [14, 15].

Pathological studies in AMAN show a relative paucity of inflammatory infiltrate with axonal destruction but this time macrophages were situated between axons and the myelin especially in the region of the node of Ranvier [16].

The pathological studies suggest that the macrophage is the instrument of nerve damage but may well be targeted to either the myelin or axon by antibodies. In AMSAN pathological changes are similar but involve both motor and ventral nerve roots [17].

## 4. Immunology

The recognition that there was an association between GBS and a variety of triggering infections strongly suggested that there must be an immunological cause for the syndrome. This was supported by the nature of the pathological changes with macrophage targeted, demyelination in at least AIP which could be used to support an antibody mediated disorder. The efficacy of plasma exchange in shortening the time taken to recover also argued for a serum factor mediating the disease. In the 1960's Melnick [18] was one of the first to publish data suggesting complement fixing antibodies in the acute phase of GBS. These studies were difficult to replicate but sensitive C1 esterase assays supported complement consumption and a role for complement in the disorder [19]. In rabbits immunisation with galactocerebroside can produce a demyelinating neuropathy, suggesting that antibodies against myelin antigens are capable of causing neuropathy [20]. The pathology of the human disease resembled the experimental model experimental allergic neuritis produced by immunising susceptible species with peripheral nerve in adjuvant. EAN can be elicited using individual proteins from myelin such as P0 and P2 and T cell lines reacting with P2 can transfer the disease [21, 22].

This stimulated numerous studies attempting to find antibodies to P2, P0, and other protein antigens in GBS but these were largely negative [23]. Antibodies recognising lipids were identified in the 1980's and increasingly recognised in certain subgroups of GBS [24]. The identification of antibodies against one of these gangliosides, GQ1b in 95% of patients with Miller Fisher Syndrome [25, 26], supported a role for such antibodies in the pathogenesis of this syndrome thought to be very closely related to GBS. Similar antibodies were also found in GBS with ophthalmoplegia and in Bickerstaff's encephalitis [27, 28]. In vitro studies of mouse hemidiaphragm preparations showed that antiGq1b monoclonals immunostained the neuromuscular junction where they fixed complement and bound in identical ways to patient serum [29]. Antiganglioside antibodies were found to be associated with AMAN [30] and were implicated in animal models of the disease in rabbits [31]. Furthermore, patients immunised with gangliosides [32] were known to develop neuropathies in certain circumstances adding to the body of evidence supporting a pathology for GBS which involved complement fixing antibodies against human gangliosides.

Although the evidence in support of antiganglioside antibodies as a cause of MFS and AMAN was strong the most common form of GBS on Western countries (AIDP) was only rarely associated with ganglioside antibodies using conventional techniques [33]. The frequency of antiganglioside antibodies increases if antibodies against complexes of more than one ganglioside are considered although there are as yet few published studies [34, 35]. These are eagerly awaited.

Antibodies against gangliosides are usually found to be of the IgG1 or IgG3 subtype that conventionally require T cell help in their production. T cells infiltrate the pathological lesion in GBS nerve and so it seems likely that they play a part in mediating antibody production. Several studies have identified raised concentrations of activated T cells in the peripheral blood among patients with GBS [36] as well as changes in regulatory T cells [37] and raised levels of T cell derived cytokines [38]. The early studies looking at T cell reactivity against protein antigens such as the P2 Protein which were implicated in EAN proved to be negative. **ϒ** **δ** T cells that are capable of recognising nonprotein antigens such as gangliosides have been isolated from GBS nerve but may be isolated from patients with vasculitis [39]. It is possible that such T cells may play a role but strong evidence is lacking. **ϒ** **δ** T cells are restricted by CD1 which is upregulated in nerve from patients with GBS [40] but no clear CD1 polymorphism is linked to GBS [41].

The clinical features of GBS are very variable and attempts have been made to correlate this with the distribution of gangliosides in different nerves [42]. There is more GQ1b in the ocular nerves which might explain the ophthalmoplegia in Miller Fisher syndrome. Similarly ventral nerve roots contain more GM1 than dorsal roots. The actual densities and accessibilities of the gangliosides in different tissues may be more important and there are studies suggesting that access to gangliosides by antibodies may differ [43].

*C. jejuni* is the best studied triggering agent for GBS and has been shown to have ganglioside like structures in the lipopolysaccharide coat of the bacterium [44–46]. Similar examples of molecular mimicry are seen with other organisms that rigger GBS such as *Haemophilus* [47] and *Cytomegalovirus* [48]. It therefore seems plausible to hypothesise that infection with one of these agents leads to antibody production which cross-reacts with gangliosides and other glycolipids leading to myelin destruction. This could occur by complement activation or by antibodies targeting macrophages via the fc receptor and leading to both conduction failure and demyelination.

For such specific antibodies to mediate disease they would need to pass through the blood nerve barrier. Studies in EAN suggest that activated T cells may open up the barrier to allow the antineural antibodies to mediate nerve damage [49, 50]. It is of course possible that breakdown in the blood nerve barrier is a nonspecific event that allows antigen specific antibodies to penetrate and mediate disease. Matrix metalloproteinases have been implicated in mediating barrier breakdown [51]. There may be specific factors about the triggering infection that increase the likelihood of immune sensitivity to a specific agent. Certain serotypes of *C. jejuni* appear more likely to produce these autoreactive antibodies perhaps by containing more neuritogenic epitopes [52, 53]. The risk of GBS after *C. jejuni* enteritis is estimated to be about 1 in 1000. This risk must be influenced by immunological genetic factors. Studies of HLA associations with GBS are generally weak [54, 55]. Only a very small number of familial cases of GBS have been described [56, 57].

Although antiganglioside antibodies are the most commonly reported antibody in GBS there are other reports of antibodies that might be pathogenic in a small number of patients. Antibodies against a protein in the node of Ranvier “neurofascin” have received recent attention with serum of 4% of patients with AIDP being positive in one recent study [58].

## 5. Neurophysiology

Neurophysiology is extremely useful in the diagnosis and definition of the subtype of GBS. Assessment early in the course of the syndrome frequently shows small action potentials, prolonged distal motor latency, delayed F waves, and conduction block [59]. Occasionally the first study is normal and a repeat study is required to document a peripheral nerve disorder. Axonal forms of the disease are characterised by reduced motor and/or sensory action potentials with denervation potentials once the acute stage of the disease is over. Neurophysiological studies carried out as part of the European IvIg and steroid trial found 69% of the studies to be consistent with AIDP with only 3% suggesting axonal pathology on studies carried out within 3 weeks of onset. Twenty-three percent of studies were equivocal at this early stage and may have gone on to be predominantly axonal [60].

## 6. Management

Supportive aspects of management have been the major factor in improving mortality in GBS with the advent of good ITU care and modern methods of ventilation. Infection, emboli, and autonomic instability are the major causes of death. Passive movement of limbs and active physiotherapy once the initial acute stage is over appear to be beneficial although it has never been subject to a controlled clinical trial.

Active immune modulation with IvIg [61] or plasma exchange [62] is the mainstay of treatment with IvIg being preferred in most circumstances due to ease of availability and greater safety in patients with unstable blood pressure and pulse. IvIg is usually given at a dose of 0.4 gm/kg for 5 days although the optimum dose has never been established. Recent studies suggest that metabolism of IvIg is faster in patients with a worse prognosis and there are studies in place to see whether a higher dose of IvIg would benefit some patients [63].

Patients that either fail to improve or exhibit a deterioration are often given a further course of IvIg although trials have yet to justify such an approach. The combination of IvIg with either steroids or plasma exchange seems to confer little benefit [64].

Better treatments of GBS are clearly needed to reduce the proportion of patients that are left disabled. Complement inhibitors such as eculizumab have been shown to be effective in animal models of Miller Fisher syndrome [65] and to be safe in man [66] but have yet to be the subject of a controlled trial. Since much of the damage to nerves occurs early in the course of the disease it may be more effective to look at chemicals capable of improving nerve regrowth and regeneration. Such neuroprotective drugs would clearly be of value in a number of diseases with a common end point of axonal damage.

## References

[1] Guillain G, Barré J, Strohl A (1916). Sur un syndrome de radiculo-nevrite avec hyperalbuminose du liquide cephalorachidien sans reaction cellulaire. Remarques sur les characteres clinique et graphique des reflexes tendinaux. *Bulletins et Memories de la Societe Medicale des Hopitaux de Paris*.

[2] Haymaker WKJ (1949). The Landry-Guillain-Barré syndrome: a clinicopathologicic report of fifty fatal cases and a critique of the literature. *Medicine*.

[3] Griffin JW, Li CY, Ho TW (1995). Guillain-Barré syndrome in northern China. The spectrum of neuropathological changes in clinically defined cases. *Brain*.

[4] McGrogan A, Madle GC, Seaman HE, De Vries CS (2009). The epidemiology of Guillain-Barré syndrome worldwide: a systematic literature review. *Neuroepidemiology*.

[5] Sejvar JJ, Baughman AL, Wise M, Morgan OW (2011). Population incidence of Guillain-Barré syndrome: a systematic review and meta-analysis. *Neuroepidemiology*.

[6] Lawn ND, Fletcher DD, Henderson RD, Wolter TD, Wijdicks EFM (2001). Anticipating mechanical ventilation in Guillain-Barré syndrome. *Archives of Neurology*.

[7] Reid AC, Draper IT (1980). Pathogenesis of papilloedema and raised intracranial pressure in Guillain-Barré syndrome. *British Medical Journal*.

[8] Colls BM (2004). Guillain-Barré syndrome and hyponatraemia. *Internal Medicine Journal*.

[9] Souayah N, Nasar A, Suri MFK, Qureshi AI (2008). National trends in hospital outcomes among patients with Guillain-é syndrome requiring mechanical ventilation. *Journal of Clinical Neuromuscular Disease*.

[10] Winer JB, Hughes RAC, Osmond C (1988). A prospective study of acute idiopathic neuropathy. I. Clinical features and their prognostic value. *Journal of Neurology Neurosurgery & Psychiatry*.

[11] Bardage C, Persson I, Ortqvist A, Bergman U, Ludvigsson JF, Granath F (2011). Neurological and autoimmune disorders after vaccination against pandemic influenza A (H1N1) with a monovalent adjuvanted vaccine: population based cohort study in Stockholm, Sweden. *BMJ*.

[12] Goddard EA, Lastovica AJ, Argent AC (1997). Campylobacter 0:41 isolation in Guillain-Barré syndrome. *Archives of Disease in Childhood*.

[13] Cresswell F, Eadie J, Longley N, Macallan D (2010). Severe Guillain-Barré syndrome following primary infection with varicella zoster virus in an adult. *International Journal of Infectious Diseases*.

[14] Asbury AK, Arnason BG, Adams RD (1969). The inflammatory lesion in idiopathic polyneuritis. Its role in pathogenesis. *Medicine*.

[15] Prineas JW (1972). Acute idiopathic polyneuritis. An electron microscope study. *Laboratory Investigation*.

[16] Griffin JW, Li CY, Macko C (1996). Early nodal changes in the acute motor axonal neuropathy pattern of the Guillain-Barré syndrome. *Journal of Neurocytology*.

[17] Griffin JW, Li CY, Ho TW (1996). Pathology of the motor-sensory axonal guillain-barré syndrome. *Annals of Neurology*.

[18] Melnick SC (1963). Thirty-eight cases of the Guillain-Barré syndrome: an immunological study. *British Medical Journal*.

[19] Koski CL, Humphrey R, Shin ML (1985). Anti-peripheral myelin antibody in patients with demyelinating neuropathy: quantitative and kinetic determination of serum antibody by complement component 1 fixation. *Proceedings of the National Academy of Sciences of the United States of America*.

[20] Saida T, Saida K, Silberberg DH, Brown MJ (1981). Experimental allergic neuritis induced by galactocerebroside. *Annals of Neurology*.

[21] Hartung H-P, Schafer B, Fierz W, Heininger K, Toyka KV (1987). Ciclosporin A prevents P2 T cell line-mediated experimental autoimmune neuritis (AT-EAN) in rat. *Neuroscience Letters*.

[22] Zhu J, Pelidou S-H, Deretzi G (2001). P0 glycoprotein peptides 56–71 and 180–199 dose-dependently induce acute and chronic experimental autoimmune neuritis in Lewis rats associated with epitope spreading. *Journal of Neuroimmunology*.

[23] Hughes RAC, Gray IA, Gregson NA (1984). Immune responses to myelin antigens in Guillain-Barré syndrome. *Journal of Neuroimmunology*.

[24] Quarles RH, Ilyas AA, Willison HJ (1986). Antibodies to glycolipids in demyelinating diseases of the human peripheral nervous system. *Chemistry and Physics of Lipids*.

[25] Chiba A, Kusunoki S, Shimizu T, Kanazawa I (1992). Serum IgG antibody to ganglioside GQ1b is a possible marker of Miller Fisher syndrome. *Annals of Neurology*.

[26] Willison HJ, Veitch J, Paterson G, Kennedy PGE (1993). Miller Fisher syndrome is associated with serum antibodies to GQ1b ganglioside. *Journal of Neurology Neurosurgery & Psychiatry*.

[27] Odaka M, Yuki N, Hirata K (2001). Anti-GQ1b IgG antibody syndrome: clinical and immunological range. *Journal of Neurology Neurosurgery & Psychiatry*.

[28] Odaka M, Yuki N, Yamada M (2003). Bickerstaff’s brainstem encephalitis: clinical features of 62 cases and a subgroup associated with Guillain-Barré syndrome. *Brain*.

[29] O’Hanlon GM, Plomp JJ, Chakrabarti M (2001). Anti-GQ1b ganglioside antibodies mediate complement-dependent destruction of the motor nerve terminal. *Brain*.

[30] Ho TW, Willison HJ, Nachamkin I (1999). Anti-GD1a antibody is associated with axonal but not demyelinating forms of Guillain-Barré syndrome. *Annals of Neurology*.

[31] Yuki N, Yamada M, Koga M (2001). Animal model of axonal Guillain-Barré syndrome induced by sensitization with GM1 ganglioside. *Annals of Neurology*.

[32] Govoni V, Granieri E, Tola MR (1997). Exogenous gangliosides and Guillain-Barré syndrome. An observational study in the Local Health District of Ferrara, Italy. *Brain*.

[33] Willison HJ, Goodyear CS (2013). Glycolipid antigens and autoantibodies in autoimmune neuropathies. *Trends in Immunology*.

[34] Kaida K, Kusunoki S (2010). Antibodies to gangliosides and ganglioside complexes in Guillain-Barré syndrome and Fisher syndrome: mini-review. *Journal of Neuroimmunology*.

[35] Kusunoki S, Kaida K-I, Ueda M (2008). Antibodies against gangliosides and ganglioside complexes in Guillain-Barré syndrome: new aspects of research. *Biochimica et Biophysica Acta*.

[36] Hu W, Janke A, Ortler S (2007). Expression of CD28-related costimulatory molecule and its ligand in inflammatory neuropathies. *Neurology*.

[37] Chi L-J, Wang H-B, Zhang Y, Wang W-Z (2007). Abnormality of circulating CD4^+^CD25^+^ regulatory T cell in patients with Guillain-Barré syndrome. *Journal of Neuroimmunology*.

[38] Nyati KK, Prasad KN, Verma A, Paliwal VK (2010). Correlation of matrix metalloproteinases-2 and -9 with proinflammatory cytokines in Guillain-Barré syndrome. *Journal of Neuroscience Research*.

[39] Ben-Smith A, Gaston JSH, Barber PC, Winer JB (1996). Isolation and characterisation of T lymphocytes from sural nerve biopsies in patients with Guillain-Barré syndrome and chronic inflammatory demyelinating polyneuropathy. *Journal of Neurology Neurosurgery & Psychiatry*.

[40] Khalili-Shiraz A, Gregson N, Hughes R (1997). CD 1 expression in human peripheral nerve of GBS patients. *Biochemical Society Transactions*.

[41] Kuijf ML, Geleijns K, Ennaji N, van Rijs W, van Doorn PA, Jacobs BC (2008). Susceptibility to Guillain-Barré syndrome is not associated with CD1A and CD1E gene polymorphisms. *Journal of Neuroimmunology*.

[42] Chiba A, Kusunoki S, Obata H, Machinami R, Kanazawa I (1997). Ganglioside composition of the human cranial nerves, with special reference to pathophysiology of Miller Fisher syndrome. *Brain Research*.

[43] Gong Y, Tagawa Y, Lunn MPT (2002). Localization of major gangliosides in the PNS: implications for immune neuropathies. *Brain*.

[44] Ang CW, Endtz HP, Jacobs BC (2000). Campylobacter jejuni lipopolysaccharides from Guillain-Barré syndrome patients induce IgG anti-GM1 antibodies in rabbits. *Journal of Neuroimmunology*.

[45] Sheikh KA, Nachamkin I, Ho TW (1998). Campylobacter jejuni lipopolysaccharides in Guillain-Barré syndrome: molecular mimicry and host susceptibility. *Neurology*.

[46] Yuki N (1997). Molecular mimicry between gangliosides and lipopolysaccharides of Campylobacter jejuni isolated from patients with Guillain-Barré syndrome and Miller Fisher syndrome. *Journal of Infectious Diseases*.

[47] Mori M, Kuwabara S, Miyake M (1999). Haemophilus influenzae has a GM1 ganglioside-like structure and elicits Guillain-Barré syndrome. *Neurology*.

[48] Khalili-Shirazi A, Gregson N, Gray I, Rees J, Winer J, Hughes R (1999). Antiganglioside antibodies in Guillain-Barré syndrome after a recent cytomegalovirus infection. *Journal of Neurology Neurosurgery & Psychiatry*.

[49] Spies JM, Pollard JD, Bonner JG, Westland KW, McLeod JG (1995). Synergy between antibody and P2-reactive T cells in experimental allergic neuritis. *Journal of Neuroimmunology*.

[50] Westland KW, Pollard JD, Sander S, Bonner JG, Linington C, McLeod JG (1999). Activated non-neural specific T cells open the blood-brain barrier to circulating antibodies. *Brain*.

[51] Créange A, Sharshar T, Planchenault T (1999). Matrix metalloproteinase-9 is increased and correlates with severity in Guillain-Barré syndrome. *Neurology*.

[52] Koga M, Gilbert M, Li J (2005). Antecedent infections in Fisher syndrome: a common pathogenesis of molecular mimicry. *Neurology*.

[53] Yuki N, Taki T, Takahashi M (1994). Penner’s serotype 4 of Campylobacter jejuni has a lipopolysaccharide that bears a GM1 ganglioside epitope as well as one that bears a GD1a epitope. *Infection and Immunity*.

[54] Winer JB, Briggs D, Welsh K, Hughes RAC (1988). HLA antigens in the Guillain-Barré syndrome. *Journal of Neuroimmunology*.

[55] Rees JH, Vaugham RW, Kondeatis E, Hughes RAC (1995). HLA-class II alleles in Guillain-Barré syndrome and miller fisher syndrome and their association with preceding Campylobacter jejuni infection. *Journal of Neuroimmunology*.

[56] MacGregor GA (1965). Familial Guillain-Barré syndrome. *The Lancet*.

[57] Saunders M, Rake M (1965). Familial Guillain-Barré syndrome. *The Lancet*.

[58] Ng JK, Malotka J, Kawakami N (2012). Neurofascin as a target for autoantibodies in peripheral neuropathies. *Neurology*.

[59] Cornblath DR (1990). Electrophysiology in Guillain-Barré syndrome. *Annals of Neurology*.

[60] Hadden RDM, Cornblath DR, Hughes RAC (1998). Electrophysiological classification of Guillain-Barré syndrome: clinical associations and outcome. *Annals of Neurology*.

[61] Hughes RA, Raphaël JC, Swan AV, van Doorn PA (2006). Intravenous immunoglobulin for Guillain-Barré syndrome. *Cochrane Database of Systematic Reviews*.

[62] Raphaël JC, Chevret S, Hughes RA, Annane D (2001). Plasma exchange for Guillain-Barré syndrome. *Cochrane Database of Systematic Reviews*.

[63] Kuitwaard K, De Gelder J, Tio-Gillen AP (2009). Pharmacokinetics of intravenous immunoglobulin and outcome in Guillain-Barré syndrome. *Annals of Neurology*.

[64] Anonymous (1997). Randomised trial of plasma exchange, intravenous immunoglobulin, and combined treatments in Guillain-Barré syndrome. *The Lancet*.

[65] Halstead SK, Humphreys PD, Zitman FMP, Hamer J, Plomp JJ, Willison HJ (2008). C5 inhibitor rEV576 protects against neural injury in an in vitro mouse model of Miller Fisher syndrome. *Journal of the Peripheral Nervous System*.

[66] Fitzpatrick AM, Mann CA, Barry S, Brennan K, Overell JR, Willison HJ (2011). An open label clinical trial of complement inhibition in multifocal motor neuropathy. *Journal of the Peripheral Nervous System*.

